# Preventive behaviors by the level of perceived infection sensitivity during the Korea outbreak of Middle East Respiratory Syndrome in 2015

**DOI:** 10.4178/epih.e2016051

**Published:** 2016-11-16

**Authors:** Soon Young Lee, Hee Jeong Yang, Gawon Kim, Hae-Kwan Cheong, Bo Youl Choi

**Affiliations:** 1 Department of Preventive Medicine and Public Health, Ajou University School of Medicine, Suwon, Korea; 2 Department of Social and Preventive Medicine, Sungkyunkwan University School of Medicine, Suwon, Korea; 3 Department of Preventive Medicine, Hanyang University College of Medicine, Seoul, Korea

**Keywords:** Middle East Respiratory Syndrome coronavirus, Epidemics, Risk reduction behavior, Cluster analysis

## Abstract

**OBJECTIVES:**

This study was performed to investigate the relationship between community residents’ infection sensitivity and their levels of preventive behaviors during the 2015 Middle East Respiratory Syndrome (MERS) outbreak in Korea.

**METHODS:**

Seven thousands two hundreds eighty one participants from nine areas in Gyeonggi-do including Pyeongtaek, the origin of the outbreak in 2015 agreed to participate in the survey and the data from 6,739 participants were included in the final analysis. The data on the perceived infection sensitivity were subjected to cluster analysis. The levels of stress, reliability/practice of preventive behaviors, hand washing practice and policy credibility during the outbreak period were analyzed for each cluster.

**RESULTS:**

Cluster analysis of infection sensitivity due to the MERS outbreak resulted in classification of participants into four groups: the non-sensitive group (14.5%), social concern group (17.4%), neutral group (29.1%), and overall sensitive group (39.0%). A logistic regression analysis found that the overall sensitive group with high sensitivity had higher stress levels (17.80; 95% confidence interval [CI], 13.77 to 23.00), higher reliability on preventive behaviors (5.81; 95% CI, 4.84 to 6.98), higher practice of preventive behaviors (4.53; 95% CI, 3.83 to 5.37) and higher practice of hand washing (2.71; 95% CI, 2.13 to 3.43) during the outbreak period, compared to the non-sensitive group.

**CONCLUSIONS:**

Infection sensitivity of community residents during the MERS outbreak correlated with gender, age, occupation, and health behaviors. When there is an outbreak in the community, there is need to maintain a certain level of sensitivity while reducing excessive stress, as well as promote the practice of preventive behaviors among local residents. In particular, target groups need to be notified and policies need to be established with a consideration of the socio-demographic characteristics of the community.

## INTRODUCTION

In May 2015, the first Korean patient was confirmed to be infected with the Middle East Respiratory Syndrome coronavirus (MERS-CoV) [1]. During the next two months, 186 patients were confirmed to be infected, 38 deaths occurred, and 16,752 people were quarantined [2]. Due to the fear of the outbreak of the disease, Korean people experienced substantial stress and changes in their daily life [3].

MERS was known to be transmitted through direct or indirect contact with the secretions of infected camels or droplets from infected people; on the other hand, it has not known there was no spread through interpersonal usual contact in their lives of community [4]. The MERS outbreak in Korea, was initiated through contact with infected people within hospitals [1]. Since the infection spread to other regions through patients who moved to hospitals in other locations, people were also concerned about the spread of the infection to their communities. As the fear of visiting hospitals and concerns about the possibility of transmission to communities emerged, the outbreak resulted in a larger social impact than the H1N1 influenza outbreak that resulted in the infection of 15,160 people and 260 deaths in 2009.

Community residents’ risk perception of public health crises tends to lead to changes in their health behaviors and induces the practice of preventive behaviors, which act as factors to control the spread of outbreaks [5-7]. On the other hand, excessive fear of infection causes unnecessary social costs [8]. In order to ensure that there is an efficient response to the outbreak of infectious disease, it is necessary to attain a balance between the continuation of daily life and the practice of preventive behaviors, through a certain level of risk perception.

Considering past infectious diseases outbreaks, including severe acute respiratory syndrome and the H1N1 influenza, several studies have been performed to assess the relationship between the perception of residents about the outbreak and their practice of preventive behaviors [5,7,9-11]. In contrast, only a few studies have been conducted to assess the perception of community residents following the recent MERS outbreak. Similar studies, although limited, have been performed on people that were quarantined or medical staffs, following the MERS outbreak [12,13]. Thus, in this study, we investigated perceived infection sensitivity among community residents and the level of preventive behaviors practiced during the 2015 outbreak of MERS in Korea. We also identified the characteristics of groups with high or low level of perceived sensitivity, by which we aimed to reveal the effect of perceived infection sensitivity on preventive behaviors.

## MATERIALS AND METHODS

### Study population

The study participants were adults aged 19 years or older, from nine areas of Gyeonggi-do (including Pyeongtaek where the first patient confirmed to be infected was identified), who agreed to participate in the Community Health Survey (CHS) in 2015. The CHS is an annual national cross-sectional study that has performed in communities since 2008. The survey is led by the Korea Centers for Disease Control and Prevention (KCDC), and is used to generate community health indices for the establishment and evaluation of regional healthcare programs [14,15].

Data were collected between September and November 2015, which was two to four months from July 6, 2015, the day of the onset of the infection in the last MERS patient. The nine areas in Gyeonggi-do were selected according to their contiguity to the outbreak area, and a self-administered questionnaire was employed. Pyeongtaek and Songtan of Pyeongtaek-si where the infected people were concentrated due to the presence of Pyeongtaek St. Mary’s Hospital (where the first infected patient was found) were classified to district 1, and Hwaseong-si, Guri-si, and Namyangju-si, where secondary and tertiary infections occurred resulting in the quarantine of many people, were grouped to district 2. Other areas in Gyeonggi-do, including Yeongtong of Suwon, Bundang of Seongnam-si, Gwangju-si, and Hanam-si were classified to district 3.

Of the 8,242 participants in the community health survey in 2015, 7,281 people (88.3%) agreed to participate in the survey, from whom a total of 6,739 people (92.6%) were finally included, except 145 cases for which survey numbers were incorrect and 397 cases for which several data were missing.

Informed consent was obtained from the participants of this study and the study protocol was approved by the institutional review board of Ajou University Hospital (AJIRB-SBR-SUR-15- 287).

### Measurements

Data used in this study included general characteristics of participants as well as response indices to health behaviors, infection sensitivity, and outbreak. General characteristics collected from the CHS included gender, age, monthly household income, education, occupation, and residential area. In addition, health behavior-related questions included questions on smoking, high-risk drinking, stress, subjective health conditions, and chronic diseases. Questions used in this survey for infection sensitivity, reliability and practice of preventive behaviors, hand washing and policy credibility were based on the study performed with community residents during the H1N1 influenza outbreak in 2009 [10], as presented in the Appendix 1. Additionally, presence or absence of experiences of isolation or quarantine resulting from MERS infection, as well as respiratory symptoms were investigated.

The four questions under perceived infection sensitivity were “Worried about being infected by MERS”; “Worried about death due to worsened health status if infected by MERS”; “Worried about MERS infection of children, the elderly or patients with chronic disease in family”; and “Worried that the nationwide epidemic of MERS will cause socioeconomic chaos.” Scores were given from one point for the least concern, to 5-point for the highest concern. Response indices to the outbreak were stress during the outbreak period, attitude toward preventive behaviors (reliability/practice of preventive behaviors and hand washing practice), and policy credibility. Stress perception during the outbreak period was measured on a 4-point scale, in which those who responded with “greatly high” or “high” were classified into the high stress level group. Each of the indices of reliability and practice of preventive behaviors was evaluated on a 5-point scale with seven questions that were selected based on the MERS preventive guidelines from the KCDC, in which those whose mean scores were at least 4-point were classified into the reliability group or the practice group. Hand washing was evaluated on a scale of 1-4, with four questions, in which those whose mean scores were at least three points were classified into the practice of hand washing group. Policy credibility was also measured on a scale of 1-5, with four questions, in which those whose mean scores were at least 4-point were classified into the policy credibility group (Appendix 1).

### Statistical analysis

In order to classify individuals who showed similar patterns of infection sensitivity into several types, cluster analysis were performed for each question in the survey. For the analysis, in order to adjust for the difference in the distribution of responses among questions, variables were standardized to make the mean 0 and the standard deviation 1 in each question, and then socio-demographic characteristics and response indices by cluster were presented as percentages. Statistical significances of the characteristics among the participants among clusters were tested with the chi-square test. To control for socio-demographic variables, multiple logistic regression analysis were performed, and then odds ratios (ORs) and 95% confidence intervals (CIs) for high stress level/reliability and practice of preventive behaviors/policy credibility of each cluster were presented relative to the non-sensitive cluster. Statistical analysis was performed by using two statistical programs, SAS version 9.3 (SAS Institute Inc., Cary, NC, USA) and SPSS 22.0 (IBM Corp., Armonk, NY, USA). A *p*-value ≤0.05 was defined as statistically significant.

## RESULTS

### Middle East Respiratory Syndrome outbreak-related sensitivity and cluster types

For the four questions on infection sensitivity, responses in the category of “strongly agree” or “agree” were considered sensitive. The highest concerns on infection sensitivity were for children or elderly family members (68.1%), followed by concerns about socioeconomic chaos (61.7%), concerns about MERS infection (58.7%), and concerns about death in case of infection (37.7%) in that order. With respect to the perceived reliability of preventive behaviors practiced by participants, frequent hand washing with soap or sanitizer was thought to be the most reliable (92.9%), while the wearing of face masks when going outside was thought to be the least reliable (74.4%). Also, frequent hand washing with soap or sanitizer was the most practiced preventive behavior (89.3%) while the wearing of face masks when going outside was the least practiced preventive behavior (47.8%), showing that items thought to be highly reliable were also being practiced mostly. For the practice of hand washing, participants who answered that they washed their hands after using restrooms accounted for the highest proportion (95.1%), and those who washed their hands with soap or hand sanitizer were 89.9%. For questions on policy credibility, the highest proportion of responses (28.7%) were for the question concerning the dissemination of information about the MERS outbreak through the mass media, while overall responses of the government to MERS outbreak had the lowest response rate (15.6%) (Table 1).

### Characteristics by perceived infection sensitivity cluster types

**Figure 1.**
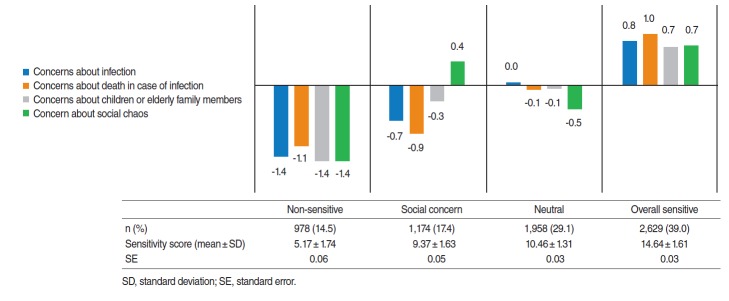
Cluster analysis results of perceived sensitivity related questions.

Cluster analysis of the questions on perceived infection sensitivity resulted in four groups. The highest rate by characteristics was found in the overall sensitive group (39.0%) with the overall highest of concerns, followed by the neutral group (29.1%) that had average level of concerns about themselves and vulnerable family members and had lower concerns about socioeconomic chaos. This was then followed by the social concern group (17.4%) that had lower concerns about themselves and family members, while they had higher concerns about socioeconomic chaos. Last was the non-sensitive group (14.5%) that had overall low sensitivity. The total score of perceived infection sensitivity was highest in the overall sensitive cluster and lowest at the non-sensitive cluster, while the neutral cluster had a higher score than the social concern cluster (Figure 1).

For general characteristics by cluster type, 62.7% of the overall sensitive group was women and those in their 30s were predominant, whereas 66.0% of the non-sensitive group was men and those in their 20s accounted for the majority. The overall sensitive group had relatively fewer high income earners, with four million Korean won or more earned as income, with many homemakers included. The non-sensitive group had a relatively higher rate of participants with menial labor jobs. In Pyeongtaek district, the non-sensitive cluster had an unexpectedly higher rate. With respect to health behaviors, the non-sensitive group had a higher proportion of smokers, risky drinkers, people with subjective good health condition, and people without chronic diseases, while there was no significant correlation with subjective stress levels. The overall sensitive group had more experiences of quarantine and active monitoring caused by MERS infection, as well as respiratory symptoms (Table 2).

### Stress level, preventive behaviors, hand washing practice and policy credibility by cluster types

Stress level during the outbreak period, reliability/practice of preventive behaviors and hand washing practice were higher in the overall sensitive group than in the non-sensitive group, while the social concern group and the neutral group showed intermediate levels. Although the high stress level group during the outbreak accounted for 37.2% of the total participants, stress levels of the group were as high as 61.4% in the overall sensitive group, while they were only 7.4% in the non-sensitive group, showing a clear distinction. Perceived reliability and practice of preventive behaviors and the practice of hand washing were also found at the highest proportion in the overall sensitive group, which was significant. Policy credibility was also high in the overall sensitive group, and low in the non-sensitive group. However, this was not significant (Table 3).

**Figure 2.**
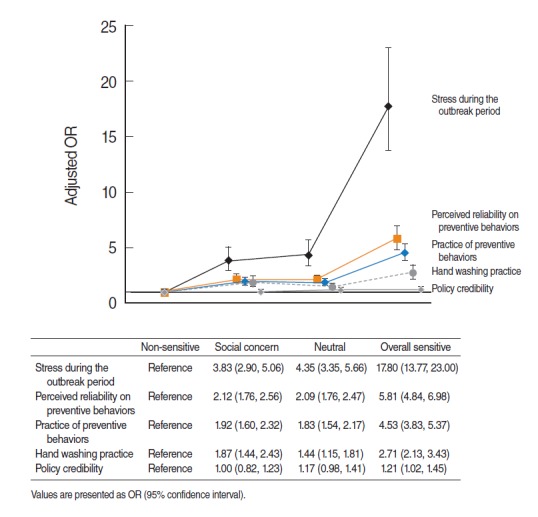
Odds ratios (ORs) of high stress level/perceived reliability and practice of preventive behaviors/hand washing/policy credibility depending on infection sensitivity type (adjusted). Gender, age, household income, occupation, residential area, smoking, drinking, normal stress level, subjective health level, morbidity of chronic disease, isolation and active monitoring and respiratory symptoms were adjusted.

Multiple logistic regression analysis were conducted to find how perceived infection sensitivity groups were associated with high stress levels, reliability and practice of preventive behaviors, the practice of hand washing and policy credibility during the outbreak. The social concern group, neutral group, and overall sensitive group had higher stress levels during the outbreak than the non-sensitive group. They were also found more significantly, to consider preventive behaviors reliable and had significantly higher rates of practicing preventive behaviors and hand washing, even after adjustment for control variables. Responses on policy credibility were significantly higher only in the overall sensitive group. In particular, the OR of high stress levels during the outbreak period in the overall sensitive group was 17.80 (95% CI, 13.77 to 23.00), indicating that the overall sensitive group had higher stress levels than the non-sensitive group (Figure 2).

## DISCUSSION

In 2015, the overall infection sensitivity of local residents during the MERS outbreak was high as shown by over 50% of responses with “concerning” or “highly concerning”, to four questions about sensitivity. This was relatively higher than that of the H1N1 influenza outbreak in 2009 conducted with community residents, in which responses to the same questions were concerns about infection (54.6%), concerns about death (35.4%), concerns about socioeconomic chaos (36.9%), and overall concerns (39.7%) [10]. In particular, concerns about social chaos or overall concerns, rather than concerns about infection or death, were higher by about 30% compared to those in the past H1N1 influenza outbreak. It is possible that respondents in this study perceived risks to be much closer because outbreak-related informations were rapidly spread through media such as the internet, along with their distrust of the government due to their inappropriate early countermeasures. The rates of practice of guidelines for prevention of the infection were 89.8% for frequent hand washing, 73.2% for not touching the face with hands, 72.4% for covering while coughing and 62.8% for avoiding places where there are many people, which were higher by 15 to 30% than those (72.2, 56.0, 35.8, and 31.6%, respectively) in the H1N1 influenza outbreak in 2009 [10]. On the other hand, response rates for policy credibility were 21.8% for preventive guidelines and the notification policy of the government, 28.7% for the policy on information delivery through mass media and 15.6% for the overall countermeasure policy of the government, which were lower by about over 10 to 20% than those (44.5, 45.7, and 24.0%, respectively) in the H1N1l influenza outbreak in 2009 [10].

Patterns of perceived infection sensitivity were different depending on socio-demographic characteristics. Clusters with relatively higher sensitivity had more women in their 30s, which was consistent with previous study results where women had a higher perceived susceptibility to infectious diseases [16,17]. In addition, the age group in their 30s mostly had young children, which was similar to previous study results in which those with children aged four years or under, practiced preventive behaviors better [11]. Men in their 20s who traditionally tend to take risks had low perceived infection sensitivity. Sensitive groups had relatively more housewives, while the non-sensitive group had relatively more people with menial or clerical jobs. On the other hand, there was no distinct difference in monthly household income or education.

Regarding health-related characteristics, the non-sensitive group had a higher rate of undesirable health behaviors such as smoking or risky drinking, whereas sensitive groups tended to have more people with poor subjective health conditions or people affected by chronic diseases. It seems that the perception that existing negative health conditions cause an increase in susceptibility to infectious disease holds true, as there was actually increased infection sensitivity. For outbreak-related characteristics, those who were subjects of quarantine or monitoring or those who experienced respiratory symptoms during the outbreak period had higher infection sensitivity, similar to previous results [7]. The Pyeongtaek area, the origin of the outbreak, tended to more residents belonging to the non-sensitive group. It might be due to the relief from the heightened fearful situation since the survey was conducted after two months of the outbreak, or a reduction of the fear of the possibility of infection, because they were finally not infected even during the actual onset of the outbreak.

This study found that participants whose sensitivity to the MERS outbreak was high tended to have a higher consideration that the practice of preventive behaviors was reliable, as well has had higher rates of the practice of preventive behaviors. These results were consistent with the existing theory that risk perception of infectious diseases should promote preventive behaviors, functioning as a factor in controlling the spread of an outbreak [18]. Consistent with results of this study, previous studies reported that as participants thought that they were more susceptible to the disease, as the corresponding disease was more severe, and as the disease was suspected to be more difficult to treat, the chances of them following guidelines were higher [5,7]. In an investigation of the H1N1 influenza outbreak of Korea in 2009, with 4,606 community residents, there were more positive health behaviors such as more frequent hand washing as H1N1-related anxiety became higher (β=1.516 for moderate and 4.103 for high compared to low, both with p<0.001). In addition, concerns about infection correlated with change in health behaviors [10].

As such, perceived sensitivity to infection was different depending on general characteristics, which subsequently led to differences in attitudes to or the practice of preventive behaviors. However, despite the absence of the characteristics that predispose to infection sensitivity, preventive behaviors against infection could be promoted by conferring a certain degree of risk perception. Communication strategies could be also developed for each group to improve the awareness of the health threat and the practice of recommended preventive behaviors. For example, vulnerable families with a low risk perception on the infection could receive tailored information to increase the awareness and perceived severity of the infection and to emphasize their practice of preventive behaviors. It would be helpful if people with low or no risk perception could be improved through the communication strategy targeting significant others such as family members and women.

In this study, higher sensitivity was accompanied by higher stress levels during the outbreak. As a countermeasure during crises of novel infectious diseases, a certain level of fear promotes attention to the infectious disease. However, the perception of Ebola in the US in 2014 caused such extensive fear in people that it led to the coining of a new word—Fearbola. This helped to promote preventive behaviors; however, it also led to social costs due to an increase in rumors [8,19]. As shown in this study, perceived sensitivity to infection increased both preventive behaviors and stress levels. In comparison to other groups, the overall sensitive group had a higher ratio of high stress levels relative to the perception of the reliability of and practice of preventive behaviors, so that it was possible that the fear of MERS not only promoted preventive behaviors, but also caused excessive stress in sensitive people.

In addition, the public tends to have higher stress levels because of uncertainty and foreign risks like MERS. In general, mass media focused on reporting information on who were to be blamed or the risks of infection, rather than accurate information about the infectious diseases, which would promote preventive behaviors [20]. Providing accurate information about the infection helps to control fears about the risks of infection. Thus, active communication of health issues from the media to the people is required. Great thought should be put into the decision of which communication method would efficiently promote an appropriate level of attention and practice of preventive behaviors among the people.

However, no matter how much actively the media communicates with the people, it would not be able to promote preventive behaviors if the government has failed to secure the transparency and credibility of infection control. In this study, similar to other studies, policy credibility was not high, which was speculated to be due to a failure in early countermeasures [21]. Policy credibility raises people’s perception of the reliability of preventive behaviors and elevates the practice of preventive behaviors. While perceived infection sensitivity also functions as a factor to increase the practice of preventive behaviors, there was no or little correlation between policy credibility and infection sensitivity in this study. In other words, policy credibility was determined by factors other than perceived infection sensitivity, and it was speculated that policy credibility affected the attitude towards preventive behaviors, independent of sensitivity. In or der to prepare a strategy to promote preventive behaviors against infection, there should be further analysis of the factors that determine infection sensitivity and policy credibility.

With respect to the interpretation and application of the results, this study had several limitations. Firstly, this study was a cross sectional research. Hence, temporal relationships between result variables such as infection sensitivity and preventive behaviors were unclear. It was possible that the reliability and practice of preventive behaviors could diminish concerns about infection, leading to reduction of sensitivity. Secondly, this study employed the use of self-administered questionnaires with questions such as those on the practice of preventive behaviors. As a result, it was possible that there could be discrepancies between the real perceptions or behaviors of participants depending on their attitude to response. Thirdly, since some participants refused to take the survey or were out of reach, there could be a bias due to the actual number of participants in the survey. Fourthly, there might be a gap between the onset of the outbreak and the time of the survey, which could cause a bias. Nevertheless, representative participants for each district were selected and a timely large-scale investigation was performed at a time not too far from the time of the outbreak; hence, the results could be considered reliable.

As international migration and travel become more rampant, infectious diseases are not just a problem for the local community or country where they occur. Local endemic diseases can become a worldwide pandemic since novel infectious diseases and recurring infectious diseases keep occurring. As a result, there will always be a fear of new emerging infectious diseases. In the event of a local outbreak of an infectious disease, it might be important to reduce excessive stress levels, while also maintaining a certain sensitivity. Moreover, community residents should be encouraged to choose and practice preventive behaviors. In particular, target groups need to be notified and policies should be established with a consideration of the socio-demographic characteristics of the community.

## Figures and Tables

**Table 1.** MERS outbreak-related perceived reliability/practice of preventive behaviors, hand washing, and policy credibility

Division	Contents of questions	n (%)
Perceived infection sensitivity	Worried about being infected by MERS	4,175 (58.7)
	Worried about death due to worsened health status if infected by MERS	2,680 (37.7)
	Worried about MERS infection of children, the elderly or patients with chronic disease in the family	4,603 (68.1)
	Worried that the nationwide epidemic of MERS will cause socioeconomic chaos	4,384 (61.7)
	Worried about MERS overall	4,727 (66.5)
Perceived reliability on/practice of preventive behaviors	Frequent hands washing with soap or sanitizer	6,608 (92.9)/6,352 (89.3)
	Not touching the eyes, the nose or the mouth with unclean hands	6,156 (86.6)/5,198 (73.2)
	Covering with tissue or a handkerchief while sneezing or coughing	6,201 (87.3)/5,130 (72.4)
	Avoiding contact with others who have a fever or respiratory symptoms	6,379 (89.8)/5,425 (76.6)
	Wearing face masks when going outside	5,286 (74.4)/3,393 (47.8)
	Avoiding places where there are many people	6,019 (84.7)/4,453 (62.8)
	Refraining from visiting patients and medical institutions	5,904 (83.0)/5,148 (72.6)
Practice of hand washing	Frequent hands washing before eating	6,576 (92.5)
	Frequent hands washing after using the restroom	6,757 (95.1)
	Frequent hands washing after returning from the outdoors	6,596 (92.8)
	Frequent hands washing with soap or hand sanitizer	6,378(89.9)
Policy credibility	The guidelines for MERS prevention and notification by the government	1,548 (21.8)
	The delivery of information about the MERS outbreak through the mass media	2,041 (28.7)
	The implementation of countermeasures against MERS infection by domestic medical institutions	1,208 (17.0)
	The countermeasure to prevent MERS from spreading (quarantine, etc.)	1.789 (25.2)
	The overall response of the government to the MERS outbreak	1,110 (15.6)

MERS, Middle East Respiratory Syndrome.

**Table 2.** General characteristics by infection sensitivity type

	Total (n = 6,739)	Non-sensitive (n = 978)	Social concern (n = 1,174)	Neutral (n = 1,958)	Overall sensitive (n = 2,629)	p-value
Gender						
Men	3,088 (45.8)	587 (60.0)	615 (52.4)	906 (46.3)	980 (37.3)	< 0.001
Women	3,651 (54.2)	391 (40.0)	559 (47.6)	1,052 (53.7)	1,649 (62.7)	
Age (yr)						
19-29	1,001 (14.9)	222 (22.7)	179 (15.2)	321 (16.4)	279 (10.6)	< 0.001
30-39	1,455 (21.6)	180 (18.4)	190 (16.2)	418 (21.3)	667 (25.4)	
40-49	1,637 (24.3)	208 (21.3)	279 (23.8)	517 (26.4)	633 (24.1)	
50-59	1,178 (17.5)	171 (17.5)	254 (21.6)	322 (16.4)	431 (16.4)	
60-69	773 (11.5)	106 (10.8)	154 (13.1)	194 (9.9)	319 (12.1)	
≥70	695 (10.3)	91 (9.3)	118 (10.1)	186 (9.5)	300 (11.4)	
Monthly household income (104 Korean won)						
<100	488 (7.4)	81 (8.4)	85 (7.4)	111 (5.7)	211 (8.2)	0.007
100-200	755 (11.4)	107 (11.1)	139 (12.0)	202 (10.5)	307 (11.9)	
200-300	1,211 (18.3)	180 (18.7)	175 (15.2)	386 (20.0)	470 (18.2)	
300-400	1,322 (19.9)	174 (18.1)	245 (21.2)	385 (19.9)	518 (20.1)	
>400	2,853 (43.0)	419 (43.6)	511 (44.2)	849 (43.9)	1,074 (41.6)	
Education						
None, elementary school graduates	784 (11.7)	99 (10.1)	137 (11.7)	220 (11.3)	328 (12.5)	0.21
Middle school graduates	504 (7.5)	74 (7.6)	99 (8.5)	122 (6.2)	209 (8.0)	
High school graduates	2,142 (31.9)	323 (33.1)	365 (31.2)	634 (32.4)	820 (31.3)	
College or higher	3,294 (49.0)	480 (49.2)	570 (48.7)	978 (50.1)	1,266 (48.3)	
Occupation						
Professional administration	1,095 (16.3)	138 (14.2)	217 (18.5)	330 (16.9)	410 (15.6)	< 0.001
Corporate job	937 (13.9)	146 (15.0)	166 (14.2)	286 (14.7)	339 (12.9)	
Sales/service	861 (12.8)	124 (12.7)	161 (13.7)	256 (13.1)	320 (12.2)	
Agriculture, forestry and fishery	126 (1.9)	27 (2.8)	30 (2.6)	24 (1.2)	45 (1.7)	
Menial labor	1,248 (18.6)	213 (21.8)	231 (19.7)	354 (18.1)	450 (17.2)	
Housewife	1,468 (21.9)	139 (14.3)	192 (16.4)	408 (20.9)	729 (27.8)	
Not employed	692 (10.3)	122 (12.5)	108 (9.2)	200 (10.3)	262 (10.0)	
Other (student/soldier)	291 (4.3)	66 (6.8)	67 (5.7)	93 (4.8)	65 (2.5)	
District						
Pyeongtaek	1,693 (25.1)	293 (30.0)	303 (25.8)	414 (21.1)	683 (26.0)	< 0.001
Adjacent area	2,140 (31.8)	291 (29.8)	354 (30.2)	659 (33.7)	836 (31.8)	
Indirect	2,906 (43.1)	394 (40.3)	517 (44.0)	885 (45.2)	1,110 (42.2)	
Smoking						
Current smoker	1,254 (18.6)	255 (26.1)	231 (19.7)	372 (19.0)	396 (15.1)	< 0.001
Past smoker	1,175 (17.4)	174 (17.8)	234 (19.9)	336 (17.2)	431 (16.4)	
Non smoker	4,309 (64.0)	549 (56.1)	709 (60.4)	1,250 (63.8)	1,801 (68.5)	
Risky drinking						
Yes	866 (12.9)	162 (16.6)	183 (15.6)	232 (11.8)	289 (11.0)	< 0.001
No	5,873 (87.1)	816 (83.4)	991 (84.4)	1,726 (88.2)	2,340 (89.0)	
Stress level						
High	1,943 (28.8)	278 (28.4)	333 (28.4)	562 (28.7)	770 (29.3)	0.92
Low	4,795 (71.2)	700 (71.6)	841 (71.6)	1,396 (71.3)	1,858 (70.7)	
Subjective health level						
Good	3,011 (44.7)	497 (50.8)	573 (48.8)	866 (44.2)	1,075 (40.9)	< 0.001
Average	2,912 (43.2)	376 (38.4)	471 (40.1)	878 (44.8)	1,187 (45.2)	
Poor	815 (12.1)	105 (10.7)	130 (11.1)	214 (10.9)	366 (13.9)	
Chronic disease morbidity						
Yes	2,893 (42.9)	400 (40.9)	524 (44.6)	802 (41.0)	1,167 (44.4)	0.04
No	3,846 (57.1)	578 (59.1)	650 (55.4)	1,156 (59.0)	1,462 (55.6)	
Quarantine or active monitoring						
Yes	51 (0.8)	7 (0.7)	5 (0.4)	8 (0.4)	31 (1.2)	0.01
No	6,682 (99.2)	971 (99.3)	1,168 (99.6)	1,947 (99.6)	2,596 (98.8)	
Respiratory symptom						
Yes	197 (2.9)	24 (2.5)	29(2.5)	39 (2.0)	105 (4.0)	< 0.001
No	6,542 (97.1)	954 (97.5)	1,145 (97.5)	1,919 (98.0)	2,524 (96.0)	

Values are presented as frequency (%).

**Table 3.** High stress level/reliability and practice of preventive behaviors/hand washing/policy credibility by infection sensitivity types

Indices	Total (n = 6,739)	Non-sensitive (n = 978)	Social concern (n = 1,174)	Neutral (n = 1,958)	Overall sensitive (n = 2,629)	p-value
Stress during the outbreak	2,641 (37.2)	72 (7.4)	289 (24.6)	555 (28.3)	1,613 (61.4)	< 0.001
Reliability of preventive behaviors	5,388 (76.4)	539 (55.6)	849 (72.9)	1,416 (72.8)	2,302 (88.3)	< 0.001
Practice of preventive behaviors	3,636 (51.9)	289 (30.2)	536 (46.2)	868 (45.2)	1,765 (68.3)	< 0.001
Practice of hand washing	6,340 (89.4)	801 (82.2)	1,042 (89.4)	1,718 (88.1)	2,437 (93.2)	< 0.001
Policy credibility	1,996 (28.1)	253 (25.9)	315 (26.9)	550 (28.1)	773 (29.5)	0.13

Values are presented as frequency (%).

## Appendix 1.

Survey questions and interpretations
