# Supplementary material for: Preventive behaviors by the level of perceived infection sensitivity during the Korea outbreak of Middle East Respiratory Syndrome in 2015
Source: Epidemiol Health. 2016 Nov 16;38:e2016051. doi: 10.4178/epih.e2016051 (PMC5309729; doi:10.4178/epih.e2016051)
Supplement: Supplementary file 1 [file epih-38-e2016051-supplementary.pdf]

2015 메르스 유행 시 지역사회 주민의 감염민감도와 예방행위

Preventive behaviors by the level of infection sensitivity of Koreans during the MERS outbreak in 2015

Abstract

OBJECTIVES: 지역사회 주민의 공중보건 위기에 대한 인식은 건강행태를 변화시키고 예방행위 실천을 유도해 유행의 확산을 제어하는 요소로 작용한다. 본 연구에서는 2015 년 한국에서 발생한 메르스 유행 시 지역 주민이 인식하는 민감도와 예방행위 수준을 파악하므로 써 감염의 민감도와 예방행위 간의 연관성을 밝히고자 하였다.

METHODS: 2015년 유행의 진원지인 평택시를 포함하여 경기도 9개 시의 지역사회건강조사 대상자(8,242명) 중 동의자7,281명을 대상으로 설문조사를 실시하였으며, 응답자 중 결측치 등을 제외한 총 6,739명의 자료를 분석에 이용하였다. 조사내용은 감염 민감도(4문항), 유행 기간 중 스트레스 인지(1문항), 예방행위에 대한 신뢰도(7문항), 예방행위 실천도(7문항), 손 씻기(4문항) 및 정책 신뢰도(5문항)이다. 감염의 민감도 4개 문항에 대해 군집분석을 실시하여, 각 군집별 유행 기간 중 스트레스,예방행위에 신뢰/실천,손 씻기 실천,정책 신뢰의 수준을 분석하였다.

RESULTS: 감염에 대한 민감도의 항목별 민감도는 소아 및 고령가족에 대한 염려(68.1%)가 가장 높았으며, 사회적 혼란에 대한 염려(61.7%), 감염에 대한 염려(58.7%), 감염 시 사망에 대한 염려(37.7%)순이었다. 감염 민감도에 대한 군집분석결과, 메르스 유행에 대한 비민감군(14.5%), 사회염려군(17.4%), 중간관심군(29.1%) 및 전반적 민감군(39.0%)으로 구분되었다. 민감도가 가장 낮은 비민감군에는 남성과 20대, 건강행태가 부정적인 경우가 많았으며, 민감도가 높을수록 30대나 노인, 여성이나 주부가 많았다. 로지스틱 회귀분석 결과, 민감도가 높은 전반적 민감군은 비민감군에 비하여 유행 기간 중 스트레스(17.80; 13.77-23.00)와 예방행위 신뢰(5.81; 4.84-6.98) 및 실천(4.53; 3.83-5.37), 손 씻기 실천율(2.71; 2.13-3.43)이 높았다.

CONCLUSIONS: 2015 년 메르스 유행 시 지역사회 주민의 감염 민감도는 성별, 연령, 직업, 건강행위와 연관성이 있으며, 감염에 대한 민감도가 높을수록 스트레스를 많이 받는 한편, 예방행위 실천에 긍정적인 영향을 미쳤다. 지역사회의 감염병 유행 시 지나친 스트레스는 줄이되 적절한 민감도를 유지하고, 지역 주민이 예방행위를 선택하고 실천하도록 하는 것이 필요하며, 특히 인구사회학적 특성을 고려한 목표집단 대상 홍보 및 정책 수립이 요구된다.

Keywords : MERS-CoV; outbreak; prevention; health behavior; cluster analysis

서론

2015 년 5 월 한국에서 첫 중동호흡기증후군 바이러스(Middle East Respiratory Syndrome Coronavirus, MERS-CoV, 이하 메르스) 감염 환자가 확진되었다 [1]. 이후 약 2 개월 동안 확진자는 186 명, 사망자는 38 명, 그리고 16,752 명에 달하는 격리자가 발생하였다 [2]. 생소한 감염병의 유행으로 인한 공포감으로 인해 한국 국민들은 상당한 스트레스와 생활의 변화를 겪었다 [3].

메르스는 주로 감염된 낙타의 분비물이나 감염자의 비말을 통한 직접 접촉 및 간접 접촉을 통해 전염되고, 지역사회의 일상적 접촉으로는 거의 발병하지 않는 것으로 알려져 있다[4]. 국내의 메르스유행 역시 주로 병원 내 사람 간 전파를 통해 이루어졌으나 [1], 병원 간 환자 이동을 통해 타 지역으로의 전파가 이루어짐에 따라 지역사회로의 감염에 대한 국민들의 우려가 발생하였다. 즉, 병원 방문에 대한 걱정과 지역사회 전파 가능성에 대한 우려가 대두되면서 2009년 15,160명의 환자와 260명의 사망자를 발생시켰던 신종인플루엔자 유행 때보다 사회적 영향력이 큰 것으로 판단된다.

지역사회 주민의 공중보건 위기에 대한 위험 인식은 건강행태를 변화시키고 예방행위 실천을 유도해 유행의 확산을 제어하는 요소로 작용한다 [5-7]. 반면, 감염에 대한 지나친 공포는 불필요한 사회적 지출을 유발하므로 [8], 효율적인 감염병 대응을 위해서는 적절한 수준의 위험 인식을 통해 일상생활의 연속선을 유지하면서 예방행위를 도입하여 균형을 이루는 것이 필요하다.

과거 SARS 와 신종인플루엔자 등의 감염병 발생에 관련하여, 주민들의 유행에 대한 인식과 예방행위 실천 등의 관련성에 대한 몇몇 연구가 진행되었다 [5, 7, 9-11]. 그러나 이번 메르스

유행에 관련된 인식에 대해서는 격리자나 의료관계자에 한정되어 있고 [12, 13], 일반 지역사회 주민들의 유행 인식에 대해서는 연구된 바가 거의 없다. 따라서 본 연구에서는 2015 년 한국에서 발생한 메르스 유행 시 지역주민이 인식하는 감염에 대한 민감도와 예방행위 수준을 파악하고, 지역사회 민감도가 높거나 낮은 집단의 특성을 파악하며, 나아가 감염에 대한 민감도가 예방행위에 미치는 영향을 밝히고자 하였다.

연구방법

Study Population

본 연구는 최초 확진자 발생지역인 평택을 포함한 경기도 9 개 지역의 2015 년 지역사회건강조사에 응한 19 세 이상 성인 남녀를 대상으로 하였다. 지역사회건강조사는 질병관리본부가 주관하여 2008 년부터 지역사회 기반으로 매년 실시하는 국가적 단면 조사로, 지역보건의료계획 수립 및 평가를 위한 각 지역의 건강지표를 생산하기 위해 실시한다 [14, 15]. 지역에 거주하는 19 세 이상 성인을 모집단으로 하며, 확률비례통계추출을 통해 전국 254 개의 지역 보건소 단위 별 약 900 명의 표본을 선정, 전문 조사자를 통한 대면 조사 방식으로 조사가 수행된다.

연구자료는 마지막 메르스 환자 발생일인 2015 년 7 월 6 일로부터 2~4 개월 이내인 2015 년 9 월~11 월에 걸쳐 수집되었다. 최초 확진자 발생지역인 평택을 포함하여 유행 지역과의 인접도에 따라 경기도 내 9 개 지역을 선정, 자기 기입식 설문지 형태로 조사를 실시하였다. 1 차 감염지인 평택성모병원이 위치해 있어 감염자가 집중적으로 분포된 평택시의 평택과 송탄은 지역 1 에, 2 차 감염 및 3 차 감염이 발생하고 격리자가 다수 발생한 화성시와 구리, 남양주시는 지역 2 로, 그 외 경기 지역인 수원영통, 성남분당, 광주, 하남은 지역 3 으로 분류하였다.

해당 지역의 2015 년 지역사회건강조사 대상자 8,242 명 중 7,281 명 (88.3%)이 조사에 동의하였고, 이 중 조사번호가 누락된 145 건과 결측 397 건을 제외한 최종 6,739 명 (92.6%) 이 연구에 포함되었다. 본 연구는 참여 대상자들에게 동의서를 얻었으며, 아주대학교병원 기관연구윤리심의위원회의 승인을 받았다 (AJIRB-SBR-SUR-15-287).

Measurements

본 연구에 사용된 자료는 일반적 특성 및 건강행위, 감염 민감도, 유행에 대한 반응 지표로 이루어져 있다. 지역사회건강조사에서 수집한 일반적 특성으로 성별, 연령, 월평균 가계 소득, 학력, 직업, 거주지역, 건강행위 관련 문항으로 흡연, 위험음주, 스트레스, 주관적 건강수준, 만성질환이 포함되어 있다. 본 조사에서 사용된 감염민감도, 예방행위신뢰도 및 실천도, 손 씻기 및 정책신뢰도에 대한 설문은 2009년 신종인플루엔자 유행 시 지역사회 주민을 대상으로 실시한 연구[10]에 기반하였으며 Appendix에 제시하였다. 추가로 메르스 감염으로 인한 격리 및 능동감시경험 여부와 호흡기증상 경험 여부를 조사하였다.

감염 민감도를 묻는 4 개 문항은 “메르스에 감염될까봐”, ”메르스에 감염되면 질병이 중해져서 사망할까봐”, “소아나 고령, 지병이 있는 가족이 메르스에 감염될까봐”, “전국적으로 메르스가 유행하여 사회경제적 혼란이 올까봐” 으로 가장 염려하지 않은 경우의 1 점부터 매우 염려한 경우의 5 점까지 순차적으로 점수를 부여하였다. 유행에 대한 반응 지표는 유행 기간 중 스트레스,예방행위에 대한 태도 (예방행위 신뢰/실천,손 씻기 실천),정책 신뢰도이다.유행 기간 중 스트레스 인지는 4 점 척도로"대단히 많이" 또는 "많이 느끼는 편"으로 응답한 경우 고스트레스군으로 분류하였다. 예방행위 신뢰 및 실천은 질병관리본부 중동호흡기증후군 예방지침을 바탕으로 선정한 7 개 문항에 대해 각각 5 점 척도로 평가하였으며 평균점수가 4 점 이상일 경우 신뢰군 또는 실천군으로 분류하였다. 손 씻기는 4 개의 문항에 대해 각각 4 점 척도로 평가하였으며 평균점수가 3 점 이상일 경우 손씻기 실천군으로 정의하였다. 정책 신뢰도도

4 개 문항에 대한 5 점 척도로 평가한 점수의 평균이 4 점 이상일 경우 정책 신뢰군으로 분류하였다(Appendix).

Statistical Analysis

본 연구에서는 각각의 개인을 유사한 감염 민감도 양상을 보이는 몇 개의 유형으로 분류하기 위하여 문항별 군집분석(Cluster Analysis)을 실시하였다. 문항별 답변의 분포 차이를 교정하기 위해 각 문항별로 변수들의 평균이 0, 표준편차가 1 이 되도록 표준화하여 분석을 실시하고, 군집에 따른 인구사회학적 특성과 반응 지표를 백분율로 나타내었다. 군집 간 대상자의 특성에 대한 통계적 유의성을 검정하기 위해 Chi-square test 를 실시하였다. 인구사회학적 변수를 통제하기 위해 다중 로지스틱 회귀 분석을 시행하여, 비민감군을 준거로 각 군집의 고스트레스/예방행위 신뢰 및 실천/정책 신뢰율에 대한 교차비와 95% 신뢰구간을 제시하였다. 자료의 통계 분석은 통계프로그램 SAS version 9.3 (SAS Institute Inc.) 및 SPSS 22.0 을 사용하여 진행하였고, p-value 가 0.05 이하인 경우를 통계적으로 유의하다고 정의하였다.

연구 결과

1. 메르스 유행 관련 민감도 및 군집 유형

메르스 유행에 대한 감염 민감도 4 문항의 매우 그렇다/그렇다를 민감하다고 정의할 때, 소아 및 고령 가족에 대한 염려가 68.1%로 가장 높았고, 사회경제적 혼란에 대한 염려(61.7%), 메르스감염으로 인한 염려(58.7%), 그리고 감염 시 사망에 대한 염려(37.7%) 순이었다. 조사대상자의 예방행위 중 가장 신뢰도가 높은 문항은 비누나 세정제로 자주 손 씻기(92.9%)였고 신뢰도가 가장 낮은 문항은 외출 시 마스크 착용하기(74.4%)였다. 예방행위 중 가장 실천도가 높았던 문항 신뢰도와 마찬가지로 비누나 세정제로 자주 손 씻기(89.3%)였으며, 실천도가 가장 낮은 문항은 외출 시 마스크 착용하기(47.8%)로 예방행위에 대한 신뢰가 높은 문항에서 실천도가 높았다. 손 씻기 실천의 경우 화장실 다녀온 후 손을 씻었다고 응답한 대상자가 95.1%로 가장 많았으며 비누나 손 세정제를 사용하여 손을 씻는 경우가 89.9%였다. 정책 신뢰도를 묻는 문항에서 대중매체를 통한 메르스 발생 정보전달이 28.7%로 가장 높았고, 전반적인 메르스 유행에 대한 정부의 대응은 15.6%로 가장 낮았다(Table 1).

-Table 1 placed here-

2. 감염 민감도군집 유형별 특성

감염 민감도 문항으로 군집 분석을 실시한 결과 4 군으로 분류되었다. 각 유형의 특징에 따라 전반적으로 염려가 높은 전반적 민감군(39.0%)이 가장 많았으며,다음으로 본인 자신과 취약한 가족에 대한 염려는 보통이며 사회경제적 혼란에 대한 염려는 다소 낮은 중간관심군(29.1%)과, 반대로 자신과 가족에 대한 염려는 낮고 사회경제적 혼란에 대한 염려가 높은 사회염려군(17.4%)그리

고 전반적으로 민감도가 낮은 비민감군(14.5%) 순이다.감염 민감도의 총점은 전반적 민감군에서 가장 높고 비민감군에서 가장 낮았는데, 사회염려군에 비해 중간관심군의 점수가 다소 높았다.

-Figure 1 placed here-

군집 유형별일반적 특성을 보면, 전반적 민감군의 62.7%가 여성이었으며, 30 대가 많은 반면, 비민감군의 60.0%가 남성으로 비교적 20 대가 많았다. 그 외에 전반적 민감군에 400 만원 이상 고소득자가 비교적 적었으며, 주부가 많이 분포하였고, 비민감군에는 직업이 기능단순노무직인 경우가 상대적으로 많았다. 지역이 평택일 경우 비민감군이 다소 높은 의외의 결과를 보였다. 건강행태에서는 비민감군에 흡연자, 위험음주자, 주관적 건강수준이 양호한 경우, 만성질환이 없는 경우가 많았으며, 평소 스트레스와는 유의한 연관성이 없었다. 전반적 민감군에서 메르스로 인한 격리 및 능동감시 경험과 호흡기증상이 경험이 많았다.

-Table 2 placed here-

3. 감염 민감도 군집 유형별스트레스, 예방행위 신뢰 및 실천율, 손 씻기 실천율, 정책신뢰도

유행 기간 중 스트레스와 예방행위 신뢰/실천, 손 씻기 실천은 비민감군에 비해 전반적 민감군에서 모두 높았으며, 사회염려군과 중간관심군은 중간 정도의 분포를 보였다.전체 대상자 중 유행 중 고스트레스군은 37.2%였으나, 전반적 민감군에서는 61.4%로 높았으며 비민감군에서는 7.4%로 낮아 큰 차이를 보였다. 예방행위신뢰율과 실천율, 손 씻기 실천율의 경우에도 전반적 민감군에서 가장 높은 양상을 보였으며 모두 유의하였다. 정책 신뢰도의 경우에도 전반적 민감군에서 높고 비민감군에서 다소 낮았으나 유의하지는 않았다.

-Table 3 placed here-

감염민감도 군집 유형이 유행기간 중 고스트레스, 예방행위 신뢰도 및 실천, 손 씻기 실천 및 정책신뢰에 미치는 영향을 다중 로지스틱 회귀 분석으로 분석한 결과, 통제변수를 보정 후에도 비민감군에 비해 사회염려군과 중간관심군, 전반적 민감군에서 유행 중 스트레스를 많이 받았고, 예방행위에 대한 신뢰와 실천율, 손 씻기 실천율이 유의하게 높았다. 정책 신뢰도는 전반적 민감군에서만 유의하게 높았다. 특히 전반적 민감군은 비민감군에 비하여 유행 기간 중 고스트레스 교차비가 17.80(95%CI 13.77-23.00)로 매우 높았다.

-Figure 2 placed here-

고찰

2015 년 메르스 유행 시 지역 주민의 감염에 대한민감도는 민감도 4 개 문항에서 염려한다 또는 매우 염려한다는 답변이 50%를 넘어 전반적으로 민감도가 높았다 .이는 2009 년 신종인플루엔자 관련지역사회 주민을 대상으로 한 동일한 설문에서 감염에 대한 염려(54.6%), 사망에 대한 염려(35.4%), 사회경제적 혼란에 대한 염려(36.9%), 전반적 염려(39.7%) 보다 상대적으로 감염 민감도가 높았다 [10].특히 감염이나 사망에 대한 우려보다는 사회적 혼란이나 전반적인 염려가 과거 신종인플루엔자 유행 때 보다 약 30% 포인트나 증가하였다.이는 미흡한 초동 대처로 인한 불신과 함께, 과거에 비해 인터넷 등 매체의 발달로 감염 관련 사건에 대한 정보가 빠르게 확산됨에 따라 위험이 더욱 가까이에 있다고 인식하였을 가능성이 있다. 예방수칙의 실천율은 자주 손 씻기 89.8%. 손으로 얼굴 만지지 않기 73.2%, 기침 시 가리기 72.4%, 사람 많은 장소 피하기 62.8%로 2009 년 신종인플루엔자 유행 시 각각 72.2%, 56.0%, 35.8%, 31.6% 보다 15%~30% 포인트씩 증가하였다[10]. 반면 정책에 대한 신뢰도는 정부의 예방지침 및 홍보 정책 21.8%, 대중매제 정보전달 정책 28.7%, 전반적 정부대응정책이 15.6%로 2009 년 신종인플루엔자 유행 시 각각 44.5%, 45.7% 그리고 24.0% 보다 약 10%~20% 포인트 이상 감소하였다[10].

감염에 대한 민감한 양상은 인구사회학적 특성에 따라 차이가 있었다. 비교적 민감도가 높은 군집에는 비교적 여자가 많고 30 대 연령층이 많았다. 이는 여성에게서 감염병에 대한 인식된 감수성이 높았던 기존 연구 결과와 일치한다 [16, 17]. 또한 30 대는 주로 어린 자녀를 양육하는 연령으로, 4 세 이하의 자녀가 있을 경우 예방행위를 더 잘 실천한다는 기존 연구결과와 유사한 양상을 보였다 [11]. 전통적으로 위험을 감수하고자 하는 성향을 가지는 20 대 남성의 경우 감염

민감도가 낮았다. 민감한 군에는 주부가 많았고, 비민감군에는 기능단순노무직이나 사무직 이 비교적 많이 속하였다. 반면 가구소득이나 학력의 경우 뚜렷한 차이를 보이지 않았다.

건강 관련 특성에서 비민감한 군에는 흡연자나 위험음주자 등 불건전한 건강행위율이 높았고, 민감군에는 주관적 건강수준이 낮거나 만성질환에 이환되어 있는 경우가 많았다. 이는 기존의 부정적인 건강 상태가 감염병에 대한 감염 감수성을 높인다는 인식이 감염에 대한 민감도를 높이는 것으로 보인다. 유행 관련 특성의 경우, 격리나 감시 대상자에 해당하거나, 유행 기간 중 호흡기 증상을 겪은 경우 감염에 대한 민감도가 높아, 기존 연구 결과와 유사하였다 [7]. 반면 거주 지역의 경우 오히려 감염의 진원지였던 평택 지역이 비민감군에 속할 가능성이 높았다. 이는 조사가 2 개월 이후에 이루어졌으므로 공포감이 사라진 상태에서 안심한 결과이거나, 실제적인 위험을 겪은 뒤에도 감염되지 않았음을 직접 체험하여서 감염에 대한 불확실성에 대한 두려움이 감소하여 민감도가 낮아진 결과일 수 있다.

본 연구 결과 메르스 유행에 대한 민감도가 높은 대상자들에게서 예방행위의 신뢰 및 실천이 높게 나타났다. 이는 감염병에 대한 위험 인식이 예방행위를 촉진시켜 유행의 확산을 제어하는 요소로 작용한다는 기존 이론과 일치한다 [18]. 기존 연구 결과 각 대상이 그 질병에 걸릴 확률이 높다고 생각될수록, 해당 질병의 심각도가 높을수록, 그 질병이 치료하기 힘들다고 생각할수록 지침에 따른 행동 변화를 보일 확률이 높아 [5, 7], 본 연구 결과와 유사하였다. 국내에서는 2009 년 신종인플루엔자 발생에 관해 국내 지역사회 주민 4,606 명을 대상으로 한 조사에서, H1N1 과 관련한 불안 정도가 높을수록 평소보다 손을 자주 씻는 등의 건강행태변화가 있었다

(low 대비 moderate, high 각각에서 β=1.516, 4.103, both $p<0.001$). 또한 감염에 대한 염려가 건강행태변화와 관련이 있었다 [10].

이와 같이 일반적 특성에 따라 감염에 대한 민감도가 차이를 보이고, 이것은 예방행위의 태도나 실천의 차이로도 이어진다. 하지만 민감하지 않은 특성을 가지고 있더라도 비민감군이 아닌 사회염려군이나 중간관심군과 같은 일정 수준의 위험 인식을 가지게 함으로서 감염에 대한 예방행위를 촉진시킬 수 있다. 본 연구에서 나타난 대상의 특성에 따라 위험 소통을 시도하고, 인식 개선을 유도해 볼 수 있다. 또한 본인의 감염 가능성에 대해 낮게 인식하더라도 취약한 가족에 대한 민감한 인식이 예방행위의 실천에 영향을 미친다는 점을 고려하여, 가족이나 주변의 가까운 여성 등을 타겟으로 한 건강 인식 메시지를 통해, 간접적으로 위험에 대한 인식을 전달하는 것이 도움이 될 수 있을 것이다

본 연구에서는 민감도가 높을수록 유행 중에 스트레스가 매우 높게 나타났다. 신종 감염병의 위기 대응에서 적정 수준의 공포감은 감염병에 대한 주의를 촉진시킨다. 하지만 Fearbola 라는 신조어를 탄생시킬 만큼 대중의 공포감이 컸던 2014 년 미국의 에볼라 인식은 예방행위를 촉진시키는 효과도 있었지만 루머 등으로 인한 사회적 비용을 요구하였다 [8, 19]. 본 연구에서 보였듯이 감염에 대한 민감도는 예방행위를 증가시키지만 스트레스도 증가시켰으며, 그 정도가 컸다는 점을 유의하여 받아들일 필요가 있다. 특히 전반적 민감군의 경우 다른 군에 비해 예방행위 신뢰/실천율 등과 대비한 고스트레스율이 더욱 높은 것으로 미루어 볼 때, 메르스에 대한 공포감이 일부 민감한 대상자들에게는 예방행위를 촉진시킴을 넘어서 과도한 스트레스를 유발했을 가능성을 고려할 수 있다.

또한 메르스와 같이 불확실하거나 생소한 위험에 대해서는 대중의 스트레스가 높게 나타난다. 일반적으로 매스미디어의 보도 방식은 감염병에 대한 정확한 정보 전달보다는 책임 소재나 위험에 대한 보도에 치중하는데, 이러한 행위도 예방행위는 촉진하나 [20], 적절한 정보 제공을 통해 감염에 대한 정확한 지식을 함양시킴으로서 실제의 수준으로 공포감을 통제한다면 보다 효율적일 것이다. 이를 위해 헬스 커뮤니케이션을 통한 적극적인 언론 및 대국민 소통이 필요하며, 어떠한 소통 방식이 적정 수준의 주의와 효과적인 예방 행위 실천을 촉진할 수 있는지에 대한 능동적인 고려가 필요할 것이다.

그러나 아무리 적극적인 언론 및 대국민 소통을 한다고 하더라도 정책에 대한 신뢰가 확보되지 않는다면 예방행위를 촉진하는 효과가 크지 않을 것이다. 본 연구에서 정책 신뢰율은 높지 않았는데, 이는 다른 조사에서도 드러난 바 있으며, 이에 대한 원인을 초동 대처 실패 등으로 꼽았다 [21]. 정책 신뢰는 예방행위의 신뢰를 높이고, 이는 예방행위의 실천율을 높이는 방향으로 이어진다. 감염 민감도 역시 예방행위의 실천을 높이는 요인으로 작용하지만 본 연구에서 정책 신뢰도와 감염 민감도와의 연관은 나타나지 않거나 매우 적게 나타났다. 곧 정책 신뢰는 감염 민감도와는 다른 요인에 따라 결정되며, 민감도와는 별개로 예방행위에 대한 태도에 영향을 미치는 것으로 추정된다. 감염예방행위의 촉진 전략을 세우기 위해서는 감염 민감도와 정책 신뢰를 결정하는 요인에 대한 추가적인 분석이 필요하다.

본 연구는 결과를 해석하고 활용함에 있어 다음과 같이 유의하여야 할 한계점들이 존재한다. 첫번째로, 단면 연구로 이루어져 감염 민감도와 예방행위 등의 결과변수 간의 시간적 선후관계가 불분명하다. 예방행위에 대한 신뢰 및 실천은 감염에 대한 우려를 감소시켜 민감도를 감소시킬

가능성이 존재한다. 두번째로, 예방행위 실천 등에 대해 스스로 답변하는 방식을 취해, 답변 태도에 따라 조사 대상자의 실제 인식이나 행태와 격차가 있는 결과를 얻었을 수 있다. 세번째로 조사에 동의하지 않거나 접촉하기 힘든 대상자들이 존재하므로 자원자 비뚤림이 발생할 수 있다. 네번째로, 유행 시점과 조사 시점의 차이가 있어 그에 따른 바이어스가 발생할 수 있다. 그럼에도 불구하고,지역을 대표하는 표본을 선정,유행이 발생한 뒤 멀지 않은 시점에서 시기적절한 조사를 대규모로 시행하여 믿을 만한 결과를 얻을 수 있었다.

국가 간의 교류가 활발해 지면서 감염성 질환의 전염은 더 이상 발생 지역이나 국가만의 문제가 아니게 되었다. 지역의 풍토병이 세계적인 유행병으로 퍼지거나, 신종 감염병과 재출현 감염병이 끊임없이 발생하고 있으며, 그 때마다 신종 감염병에 대한 공포는 계속 발생할 것이다. 지역사회의 감염병 유행 시 지나친 스트레스는 줄이되 적절한 민감도를 유지하고, 지역 주민이 예방행위를 선택하고 실천하도록 하는 것이 필요하며, 특히 인구사회학적 특성을 고려한 목표집단 대상 홍보 및 정책 수립이 요구된다.

* 본 연구는 경기도 감염병 관리본부 정책연구용역사업의 연구비(2015-001)를 지원받아 수행되었습니다.

References

1.Ki M. 2015 MERS outbreak in Korea: hospital-to-hospital transmission. Epidemiol Health 2015;37: e2015033

2.Korea Centers for Disease Control and Prevention Middle East respiratory syndrome information 2015. [cited 2016 Oct 1]. Available from: http://mers.go.kr/mers/html/jsp/main.jsp (Korean). Available from:

3.Dyer O. South Korea scrambles to contain MERS virus. BMJ : British Medical Journal 2015;350:h3095

4.de Groot RJ, Baker SC, Baric RS, Brown CS, Drosten C, Enjuanes L, et al. Middle East respiratory syndrome coronavirus (MERS-CoV): announcement of the Coronavirus Study Group. J Virol 2013;87:7790-7792.

5.Tang CS-k, Wong C-y. Factors influencing the wearing of facemasks to prevent the severe acute respiratory syndrome among adult Chinese in Hong Kong. Prev Med 2004;39:1187-1193.

6.Rosenstock IM, Strecher VJ, Becker MH. The health belief model and HIV risk behavior change. Preventing AIDS: Springer; 1994, p. 5-24.

7.Leung G, Lam T, Ho L, Ho S, Chan B, Wong I, et al. The impact of community psychological responses on outbreak control for severe acute respiratory syndrome in Hong Kong. J Epidemiol Community Health 2003;57:857-863.

8.Kinsman J. “A time of fear”: local, national, and international responses to a large Ebola outbreak in Uganda. Globalization and health 2012;8:1.

9.Lau J, Yang X, Tsui H, Kim J. Monitoring community responses to the SARS epidemic in Hong Kong: from day 10 to day 62. J Epidemiol Community Health 2003;57:864-870.

10.Kim SJ, Han JA, Lee T-Y, Hwang T-Y, Kwon K-S, Park KS, et al. Community-Based Risk Communication Survey: Risk Prevention Behaviors in Communities during the H1N1 crisis, 2010. Osong public health and research perspectives 2014;5:9-19.

11.Rubin GJ, Amlôt R, Page L, Wessely S. Public perceptions, anxiety, and behaviour change in relation to the swine flu outbreak: cross sectional telephone survey. BMJ 2009;339:b2651.

12.Mackay IM, Arden KE. Middle East respiratory syndrome: an emerging coronavirus infection tracked by the crowd. Virus Res 2015;202:60-88.

13.Khan MU, Shah S, Ahmad A, Fatokun O. Knowledge and attitude of healthcare workers about middle east respiratory syndrome in multispecialty hospitals of Qassim, Saudi Arabia. BMC Public Health 2014;14:1.

14.Kim YT, Choi BY, Lee KO, Kim H, Chun JH, Kim SY, et al. Overview of Korean Community Health Survey [in Korean]. J Korean Med Assoc 2012;55:74.

15.Kang YW, Ko YS, Kim YJ, Sung KM, Kim HJ, Choi HY, et al. Korea Community Health Survey Data Profiles. Osong Public Health and Research Perspectives 2015;6:211-217.

16.Park J-H, Cheong H-K, Son D-Y, Kim S-U, Ha C-M. Perceptions and behaviors related to hand hygiene for the prevention of H1N1 influenza transmission among Korean university students during the peak pandemic period. BMC Infect Dis 2010;10:1.

17.Joseph TFL, Xilin Y, Ellie P, Tsui HY, Eric W, Yun Kwok W. SARS-related Perceptions in Hong Kong. Emerging Infectious Disease journal 2005;11:417.

18.Brewer NT, Weinstein ND, Cuite CL, Herrington Jr JE. Risk perceptions and their relation to risk behavior. Ann Behav Med 2004;27:125-130.

19.James JJ. Fearbola. Disaster Med Public Health Prep 2014;8:465-466.

20.Fogarty AS, Holland K, Imison M, Blood RW, Chapman S, Holding S. Communicating uncertainty - how Australian television reported H1N1 risk in 2009: a content analysis. BMC Public Health 2011;11:181.

21.Kim EY, Liao Q, Yu ES, Kim JH, Yoon SW, Lam WWT, et al. Middle East respiratory syndrome in South Korea during 2015: Risk-related perceptions and quarantine attitudes. Am J Infect Control 2016(in press)

Figure 1. 민감도 관련 문항 군집분석 결과

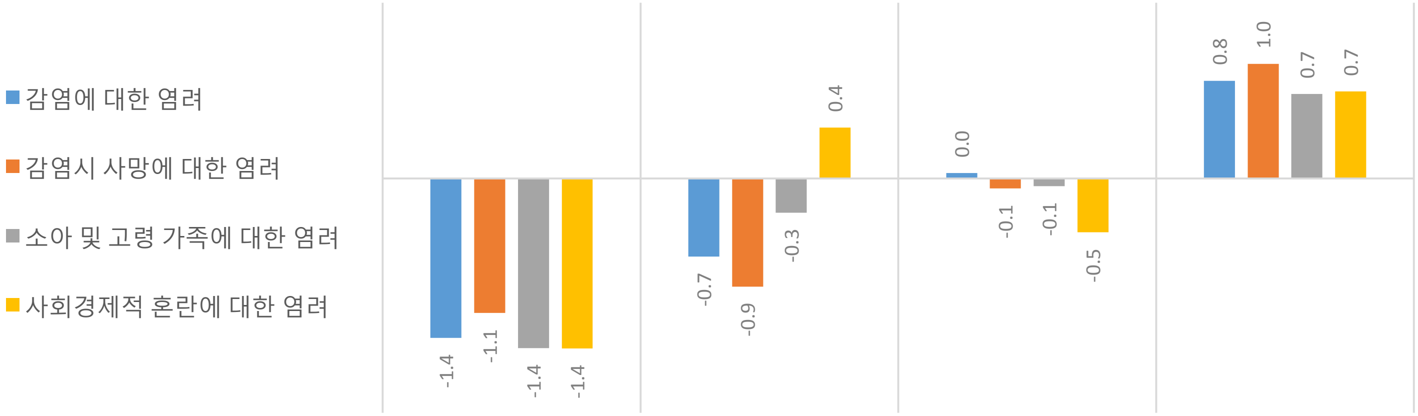

	비민감군	사회염려군	중간관심군	전반적 민감군
n	978	1,174	1,958	2,629
%	14.5	17.4	29.1	39.0
Sensitivity score (mean)	5.17	9.37	10.46	14.64
S.D.	1.74	1.63	1.31	1.61
S.E.	0.06	0.05	0.03	0.03

Figure 2. 감염 민감도 유형에 따른 고스트레스/예방행위 신뢰/실천/손 씻기/정책 신뢰율 교차비 (adjusted)

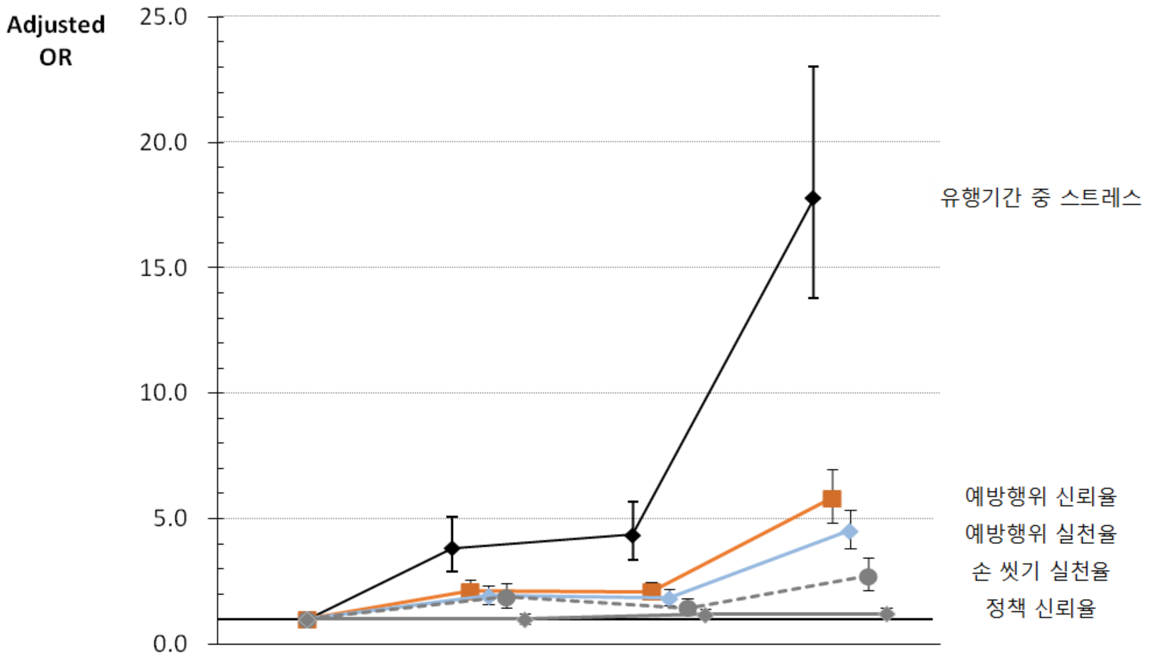

	비민감군	사회염려군	중간관심군	전반적 민감군
유행기간 중 스트레스	reference	3.83 (2.90-5.06)	4.35 (3.35-5.66)	17.80 (13.77-23.00)
예방행위 신뢰율	reference	2.12 (1.76-2.56)	2.09 (1.76-2.47)	5.81 (4.84-6.98)
예방행위 실천율	reference	1.92 (1.60-2.32)	1.83 (1.54-2.17)	4.53 (3.83-5.37)
손 씻기 실천율	reference	1.87 (1.44-2.43)	1.44 (1.15-1.81)	2.71 (2.13-3.43)
정책 신뢰율	reference	1.00 (0.82-1.23)	1.17 (0.98-1.41)	1.21 (1.02-1.45)

OR, odds ratio; CI, confidence interval.

성별,연령, 가구소득,직업,거주지역,흡연,음주,평소 스트레스,주관적 건강수준, 만성질환이환, 격리 및 능동감시 여부,호흡기증상 경험 여부를 보정함

Table 1. 메르스 유행관련 예방행위 신뢰도/실천도 및 손씻기, 정책신뢰도

구분	설문내용	N(%)
감염 민감도	메르스에 감염될까봐	4,175(58.7)
	메르스에 감염되면 질병이 중해져서 사망할까봐	2,680(37.7)
	소아나 고령, 지병이 있는 가족이 메르스에 감염될까봐	4,603(68.1)
	전국적으로 메르스가 유행하여 사회경제적 혼란이올까봐	4,384(61.7)
	전반적으로 메르스 때문에	4,727(66.5)
예방수칙 신뢰/실천	비누나 세정제로 자주 손씻기	6,608(92.9)/6,352(89.3)
	씻지 않은 손으로 눈, 코, 입을 만지지 말기	6,156(86.6)/5,198(73.2)
	재채기나 기침시 휴지나 손수건으로 가리기	6,201(87.3)/5,130(72.4)
	발열, 호흡기 증상이 있는 타인과의 접촉을 피하기	6,379(89.8)/5,425(76.6)
	외출 시 마스크 착용하기	5,286(74.4)/3,393(47.8)
	사람들이 많이 모이는 장소를 피하기	6,019(84.7)/4,453(62.8)
	병문안 및 의료기관 방문을 자제하기	5,904(83.0)/5,148(72.6)
손씻기 실천	식사하기 전 얼마나 자주 손을 씻었습니까?	6,576(92.5)
	화장실 다녀온 후 얼마나 자주 손을 씻었습니까?	6,757(95.1)
	외출 후 집에 돌아왔을 때 얼마나 자주 손을 씻었습니까	6,596(92.8)
	비누나 손 세정제를 얼마나 자주 사용하였습니까?	6,378(89.9)
정책신뢰도	정부의 메르스 예방 지침과 홍보는 어떠하였습니까?	1,548(21.8)
	대중매체를 통한 메르스 발생 정보 전달은 어떠하였습니까?	2,041(28.7)
	국내 의료기관의 메르스 감염 대응 능력은 어떠하였습니까?	1,208(17.0)
	메르스 확산 방지 대책(격리조치 등)은 어떠하였습니까?	1.789(25.2)
	전반적인 메르스 유행에 대한 정부의 대응은 어떠하였습니까?	1,110(15.6)

Table 2. 감염 민감도 유형에 따른 일반적 특성

	합계 n=6,739	비민감군 n=978	사회염려군 n=1,174	중간관심군 n=1,958	전반적 민감군 n=2,629	p
성별						
남	3,088 (45.8)	587 (60.0)	615 (52.4)	906 (46.3)	980 (37.3)	<0.001
여	3,651 (54.2)	391 (40.0)	559 (47.6)	1,052 (53.7)	1,649 (62.7)	
연령						
19-29 세	1,001 (14.9)	222 (22.7)	179 (15.2)	321 (16.4)	279 (10.6)	<0.001
30-39 세	1,455 (21.6)	180 (18.4)	190 (16.2)	418 (21.3)	667 (25.4)	
40-49 세	1,637 (24.3)	208 (21.3)	279 (23.8)	517 (26.4)	633 (24.1)	
50-59 세	1,178 (17.5)	171 (17.5)	254 (21.6)	322 (16.4)	431 (16.4)	
60-69 세	773 (11.5)	106 (10.8)	154 (13.1)	194 (9.9)	319 (12.1)	
70 세이상	695 (10.3)	91 (9.3)	118 (10.1)	186 (9.5)	300 (11.4)	
월가구소득						
~100 만원	488 (7.4)	81 (8.4)	85 (7.4)	111 (5.7)	211 (8.2)	0.007
100-200 만원	755 (11.4)	107 (11.1)	139 (12.0)	202 (10.5)	307 (11.9)	
200-300 만원	1,211 (18.3)	180 (18.7)	175 (15.2)	386 (20.0)	470 (18.2)	
300-400 만원	1,322 (19.9)	174 (18.1)	245 (21.2)	385 (19.9)	518 (20.1)	
400 만원~	2,853 (43.0)	419 (43.6)	511 (44.2)	849 (43.9)	1,074 (41.6)	
학력						
무학,초졸	784 (11.7)	99 (10.1)	137 (11.7)	220 (11.3)	328 (12.5)	0.212
중졸	504 (7.5)	74 (7.6)	99 (8.5)	122 (6.2)	209 (8.0)	
고졸	2,142 (31.9)	323 (33.1)	365 (31.2)	634 (32.4)	820 (31.3)	
대학교이상	3,294 (49.0)	480 (49.2)	570 (48.7)	978 (50.1)	1,266 (48.3)	
직업						
전문행정관리직	1,095 (16.3)	138 (14.2)	217 (18.5)	330 (16.9)	410 (15.6)	<0.001
사무직	937 (13.9)	146 (15.0)	166 (14.2)	286 (14.7)	339 (12.9)	
판매/서비스직	861 (12.8)	124 (12.7)	161 (13.7)	256 (13.1)	320 (12.2)	
농림어업	126 (1.9)	27 (2.8)	30 (2.6)	24 (1.2)	45 (1.7)	
기능단순노무직	1,248 (18.6)	213 (21.8)	231 (19.7)	354 (18.1)	450 (17.2)	
주부	1,468 (21.9)	139 (14.3)	192 (16.4)	408 (20.9)	729 (27.8)	
무직	692 (10.3)	122 (12.5)	108 (9.2)	200 (10.3)	262 (10.0)	
기타(학생/군인)	291 (4.3)	66 (6.8)	67 (5.7)	93 (4.8)	65 (2.5)	
지역						
지역 1_평택	1,693 (25.1)	293 (30.0)	303 (25.8)	414 (21.1)	683 (26.0)	<0.001
지역 2_근접지역	2,140 (31.8)	291 (29.8)	354 (30.2)	659 (33.7)	836 (31.8)	

지역 3_간접	2,906 (43.1)	394 (40.3)	517 (44.0)	885 (45.2)	1,110 (42.2)	
흡연						
흡연	1,254 (18.6)	255 (26.1)	231 (19.7)	372 (19.0)	396 (15.1)	<0.001
과거흡연	1,175 (17.4)	174 (17.8)	234 (19.9)	336 (17.2)	431 (16.4)	
비흡연	4,309 (64.0)	549 (56.1)	709 (60.4)	1,250 (63.8)	1,801 (68.5)	
위험음주						
위험음주	866 (12.9)	162 (16.6)	183 (15.6)	232 (11.8)	289 (11.0)	<0.001
미해당	5,873 (87.1)	816 (83.4)	991 (84.4)	1,726 (88.2)	2,340 (89.0)	
평소 스트레스						
고스트레스	1,943 (28.8)	278 (28.4)	333 (28.4)	562 (28.7)	770 (29.3)	0.919
저스트레스	4,795 (71.2)	700 (71.6)	841 (71.6)	1,396 (71.3)	1,858 (70.7)	
주관적건강수준						
양호	3,011 (44.7)	497 (50.8)	573 (48.8)	866 (44.2)	1,075 (40.9)	<0.001
보통	2,912 (43.2)	376 (38.4)	471 (40.1)	878 (44.8)	1,187 (45.2)	
비양호	815 (12.1)	105 (10.7)	130 (11.1)	214 (10.9)	366 (13.9)	
만성질환이환						
예	2,893 (42.9)	400 (40.9)	524 (44.6)	802 (41.0)	1,167 (44.4)	0.038
아니오	3,846 (57.1)	578 (59.1)	650 (55.4)	1,156 (59.0)	1,462 (55.6)	
격리 및 능동감시						
미해당	6,682 (99.2)	971 (99.3)	1,168 (99.6)	1,947 (99.6)	2,596 (98.8)	0.011
격리 및 감시대상	51 (0.8)	7 (0.7)	5 (0.4)	8 (0.4)	31 (1.2)	
호흡기증상						
없음	6,542 (97.1)	954 (97.5)	1,145 (97.5)	1,919 (98.0)	2,524 (96.0)	<0.001
증상있음	197 (2.9)	24 (2.5)	29 (2.5)	39 (2.0)	105 (4.0)	

Values are presented as frequency (%).

Table 3. 감염 민감도 유형에 따른 고스트레스/예방행위 신뢰/실천/손 씻기/정책 신뢰율

지표	합계<br>n=6,739	비민감군<br>n=978	사회염려군<br>n=1,174	중간관심군<br>n=1,958	전반적 민감군<br>n=2,629	p
유행기간 중 스트레스	2,641 (37.2)	72 (7.4)	289 (24.6)	555 (28.3)	1,613 (61.4)	<0.001
예방행위 신뢰율	5,388 (76.4)	539 (55.6)	849 (72.9)	1,416 (72.8)	2,302 (88.3)	<0.001
예방행위 실천율	3,636 (51.9)	289 (30.2)	536 (46.2)	868 (45.2)	1,765 (68.3)	<0.001
손 씻기 실천율	6,340 (89.4)	801 (82.2)	1,042 (89.4)	1,718 (88.1)	2,437 (93.2)	<0.001
정책 신뢰율	1,996 (28.1)	253 (25.9)	315 (26.9)	550 (28.1)	773 (29.5)	0.129

Values are presented as frequency (%)
