# Supplementary material for: Preventive behaviors by the level of perceived infection sensitivity during the Korea outbreak of Middle East Respiratory Syndrome in 2015
Source: Epidemiol Health. 2016 Nov 16;38:e2016051. doi: 10.4178/epih.e2016051 (PMC5309729; doi:10.4178/epih.e2016051)
Supplement: Supplementary file 2 [file epih-38-e2016051-app1.pdf]

Appendix 1. Survey questions and interpretations

Category		Quarantions/choices and scoring
Infection sensitivity	Question items	How much did you worry about the items below during the MERS outbreak period?<br>Worried about being infected by MERS<br>Worried about death due to worsening of the disease if infected by MERS<br>Worried about MERS infection of children, the elderly or patients with chronic disease in the family<br>Worried that the nationwide epidemic of MERS will cause socioeconomic chaos
	Choices and scores	Strongly agree: 5 points<br>Agree: 4 points<br>Average: 3 points<br>Disagree: 2 points<br>Strongly disagree: 1 point
Reliability of preventive behaviors	Question items	How much do you think the following preventive guidelines can reduce the risk of MERS infection?<br>Frequent hand washing with soap or sanitizer<br>Not touching the eyes, the nose or the mouth with unclean hands<br>Covering with tissue or a handkerchief while sneezing or coughing<br>Avoiding contact with others who have a fever or respiratory symptoms<br>Wearing face masks when going outside<br>Avoiding places where there are many people<br>Refraining from visiting patients and medical institutions
	Choices and scores	Strongly agree: 5 points<br>Agree: 4 points<br>Average: 3 points<br>Disagree: 2 points<br>Strongly disagree: 1 point
Practice of preventive behaviors	Question items	How well did you practice the following guidelines for prevention during the MERS outbreak period?<br>Frequent hand washing with soap or sanitizer<br>Not touching the eyes, the nose or the mouth with unclean hands<br>Covering with tissue or a handkerchief while sneezing or coughing<br>Avoiding contact with others who have a fever or respiratory symptoms<br>Wearing face masks when going outside<br>Avoiding places where there are many people<br>Refraining from visiting patients and medical institutions
	Choices and scores	Extremely well-5 points<br>Good-4 points<br>Average-3 points<br>Bad-2 points<br>Not at all-1 point
Practice of hand washing	Question items	Followings are questions about hand washing during the MERS outbreak period.<br>How often did you wash your hands before eating?<br>How often did you wash your hands after using the restroom?<br>How often did you wash your hands after returning from the outdoors?<br>How often did you wash your hands with soap or hand sanitizer?
	Choices and scores	Always: 4 points<br>Frequently: 3 points<br>Sometimes: 2 points<br>Hardly: 1 point
Policy credibility	Question items	Do you think the following notifications and policies for the MERS outbreak were appropriate?<br>How did you find guidelines for MERS prevention and notification by the government?<br>How did you find the delivery of information about the MERS outbreak through the mass media?<br>How did you find the implementation of countermeasures against MERS infection by domestic medical institutions?<br>How did you find the countermeasure to prevent MERS from spreading (quarantine, etc.)?
	Choices and scores	Highly appropriate: 5 points<br>Appropriate: 4 points<br>Average: 3 points<br>Inappropriate: 2 points<br>Extremely inappropriate: 1 point

MERS, Middle East Respiratory Syndrome.
